# Synthesis and Characterization of PEG-*b*-1-Vinyl Imidazole Diblock Copolymers and Their Preliminary Evaluation for Biomedical Applications

**DOI:** 10.3390/polym17121608

**Published:** 2025-06-09

**Authors:** Elina N. Kitiri, Antonio Shegani, Ioannis Kopanos, Nektarios Pirmettis, Charalampos Triantis, Maria Rikkou-Kalourkoti

**Affiliations:** 1 Department of Pharmacy, Frederick University, Nicosia 1036, Cyprus; hsc.ke@frederick.ac.cy (E.N.K.); giannhskp@gmail.com (I.K.); hsc.tc@frederick.ac.cy (C.T.); 2 Institute of Nuclear & Radiological Sciences & Technology, Energy & Safety, National Centre for Scientific Research “Demokritos”, 15310 Athens, Greece; ant.she@hotmail.gr (A.S.); npirmettis@outlook.com (N.P.)

**Keywords:** diblock copolymers, reversible addition–fragmentation chain transfer polymerization, polymeric nanocarriers, Technetium-^99m^ labeling

## Abstract

Amphiphilic diblock copolymers comprising polyethylene glycol (PEG) and 1-vinyl imidazole (VIM) were synthesized using reversible addition–fragmentation chain transfer (RAFT) polymerization. The study focused on the synthesis of well-defined nanostructures with tunable composition and their functional modification for biomedical applications. The successful polymerization of PEG-*b*-PVIM diblock copolymers was confirmed via ^1^H NMR spectroscopy, and their molecular weights were analyzed using gel permeation chromatography (GPC). The copolymers exhibited pH-responsive behavior, with effective pK values of approximately 4.2. To facilitate radiolabeling and in vivo tracking, a post-polymerization modification enabled the conjugation of a 1,4,7-Triazacyclononane-1,4,7-triacetic acid (NOTA) chelator via aminolysis. The final conjugates were purified and characterized, confirming successful functionalization. These findings highlight the potential of PEG_x_-*b*-PVIM_y_ diblock copolymers for biomedical applications.

## 1. Introduction

Polymeric nanocarriers have gained significant attention for biomedical applications owing to their structural tunability, pH-responsiveness, and ability to form stable nanostructures [1,2]. Among these, amphiphilic block copolymers are particularly advantageous, as they self-assemble into well-defined nanoscale structures such as micelles. These micellar systems feature a hydrophilic outer shell, which enhances stability in physiological environments, and a hydrophobic core, which can host hydrophobic guest molecules or functional moieties within the core [3].

Poly(ethylene glycol) (PEG) is widely used in biomedical formulations due to its hydrophilicity, low immunogenicity, and proven safety profile [4,5,6,7]. The incorporation of PEG in polymeric nanocarriers reduces protein adsorption, enhances colloidal stability, and prevents rapid clearance by the immune system, thereby influencing their biodistribu istion and interaction with biological systems [6,7]. In addition, the size and molecular composition of nanocarriers significantly influence their pharmacokinetics and biodistribution. Nanocarriers in the 10–200 nm range demonstrate optimal circulation behavior, avoiding rapid renal clearance while taking advantage of the enhanced permeability and r is notetention (EPR) effect for passive targeting [8,9].

The incorporation of imidazole moieties into polymeric structures has also attracted interest in biomedical applications due to their distinct physicochemical properties. Imidazole, a nitrogen-containing heterocyclic ring, plays a key role in biological interactions, including enzymatic catalysis and molecular recognition [10]. Synthetic polymers bearing imidazole groups have been investigated for drug delivery applications, as they exhibit hydrogen bonding capabilities, pH responsiveness, and metal coordination properties, which make them attractive candidates for stimuli-responsive and functional polymer architectures [11,12,13,14,15]. Various polymerization techniques, including free radical polymerization, nitroxide-mediated polymerization (NMP), and group transfer polymerization (GTP) [13], have been explored for the synthesis of imidazole-functionalized polymers. However, reversible addition–fragmentation chain transfer (RAFT) polymerization [16] has emerged as a preferred approach due to its ability to precisely control molecular weight and copolymer composition, enabling the fabrication of well-defined amphiphilic materials [15,17,18,19].

However, to the best of our knowledge, PEG-1-vinyl imidazole (PEG-*b*-VIM) diblock copolymers have not been previously reported, nor has their use been explored in the context of radiolabeling. The synthesis of such amphiphilic copolymers is challenging due to the polarity and coordinating nature of the imidazole monomer, which can interfere with controlled polymerization [12]. Furthermore, while imidazole-based copolymers have been explored for biomedical use, their potential in radiometal chelation and in vivo tracking remains underexplored.

In this study, we report the first example of PEG-*b*-1-vinyl imidazole (VIM) diblock copolymers synthesized using RAFT polymerization. The resulting copolymers were characterized by ^1^H NMR spectroscopy to confirm their molecular composition, and their polymerization behavior was systematically evaluated. Furthermore, a post-polymerization modification via aminolysis enabled the conjugation of a NOTA chelator to study their pharmacokinetics.

## 2. Experimental Section

Materials and Methods. All chemical reagents used in this study were of analytical grade and obtained from Sigma-Aldrich Germany, Acros Organics Leicestershire, UK, and Fluka, Roumania. Unless otherwise stated, they were used as received without further purification. High-performance liquid chromatography (HPLC)-grade solvents were used for all analytical procedures and degassed using a continuous helium flux. Deuterated solvents, including deuterated chloroform (CDCl_3_, 99.8%), deuterated dimethyl sulfoxide (d^6^-DMSO, 99.9%), and deuterium oxide (D_2_O), were purchased from Merck, Germany. Tetrahydrofuran (THF, 99.8%) was sourced from Labscan Analytical Science Gliwice, Poland. For the synthesis of the Macro-DMPA chain transfer agent, poly(ethylene oxide) (PEO) with molecular weights Mn = 750 g/mol, Mn = 2000 g/mol, and Mn = 5000 g/mol, 4-dimethylaminopyridine (DMAP), dicyclohexylcarbodiimide (DCC), 2-Dodecylthiocarbonylthio-2-methylpropanoic acid (DMPA), tripotassium phosphate, and carbon disulfide were used along with 1-dodecanethiol, all obtained from Sigma-Aldrich, Germany. The polymerization of VIM was carried out using reversible addition–fragmentation chain transfer (RAFT) polymerization, with 2,2′-Azobis(2-methylpropionitrile) (AIBN, 98%) as the radical initiator and glacial acetic acid (100%) as the reaction medium. 1-Vinyl Imidazole (VIM, >99%) was purified by passage through a basic alumina column, followed by stirring over calcium hydride to eliminate residual moisture and protic contaminants. A small quantity of 2,2-diphenyl-1-picrylhydrazyl hydrate (DPPH, 95%) was added as a radical inhibitor. The purified VIM was stored at 5 °C and vacuum-distilled immediately prior to use to ensure high purity.

To improve the biomedical applicability of the copolymers, post-polymerization modification was performed via aminolysis. Diazabicyclo [5.4.0]undec-7-ene (DBU) was used to remove the trithiocarbonate end-group, facilitating the conjugation of a NOTA chelator (2,2′-(7-(2-((2-(2,5-dioxo-2,5-dihydro-1H-pyrrol-1-yl)ethyl)amino)-2-oxoethyl)-1,4,7-triazonane-1,4-diyl)diacetic acid, Maleimide-NOTA, sourced from Chematech, Dijon France) for radiolabeling.

For polymer radiolabeling, sodium pertechnetate (Na^99m^TcO_4_) was obtained in physiological saline from a commercial ^99^Mo/^99m^Tc generator (Ultra-Technekow™ V4 Generator, Curium Pharma, Petten, The Netherlands). The precursor *fac*-[^99m^Tc(CO)_3_(H_2_O)_3_]^+^ was synthesized in-house using a reaction kit containing 5.5 mg NaBH_4_, 4 mg Na_2_CO_3_, and 20 mg Na-K tartrate. After purging with carbon monoxide gas, Na^99m^TcO_4_ was introduced following established literature procedures to obtain the final radiolabeled species [20].

All chromatographic analyses were performed using a Waters 600 system (Waters, Belgium) equipped with a Waters 2487 Dual λ absorbance detector and a Gabi gamma detector (Raytest, Germany). Separations were conducted on a Macherey-Nagel Nucleosil RP-C18 column (10 μm, 250 × 4 mm) using a binary gradient system. The mobile phase consisted of water with 0.1% trifluoroacetic acid (TFA) (Phase A) and methanol with 0.1% TFA (Phase B). The gradient began at 90% A (10% B) for 1 min, then linearly shifted to 10% A (90% B) over the next 9 min, maintaining this composition for an additional 10 min.

**Synthesis of 2-Dodecylthiocarbonylthio-2-methylpropanoic Acid (DMPA).** The chain transfer agent was synthesized following established protocols [21]. In a 250 mL round-bottom flask, 8.39 g of tripotassium phosphate (39.52 mmol), 9.5 mL of 1-dodecanethiol (8 g, 39.52 mmol), and 50 mL of acetone were mixed and stirred for 30 min. Following this, 6.5 mL of carbon disulfide (107.79 mmol) was added, leading to a visible yellow coloration of the solution. After an additional 30 min, 6 g of 2-bromo-2-methylpropionic acid (35.93 mmol) was introduced, and the reaction was maintained under stirring for 24 h. The reaction mixture was then filtered, acidified using 1 M HCl, and extracted with DCM. The organic layers were dried over anhydrous magnesium sulfate, and the crude product was purified via column chromatography using a hexane/ethyl acetate (90:10) mobile phase. The final product, obtained as a yellow solid in 53.4% yield, was structurally confirmed using ^1^H-NMR spectroscopy.

**Synthesis of Macro PEG-DMPA.** Three variations of Macro-DMPA were prepared via esterification of poly(ethylene glycol) methyl ether (m-PEG-OH) with different molecular weights (750 g/mol (DP = 16), 2000 g/mol (DP = 45), and 5000 g/mol (DP = 114)). For the synthesis of m-PEG_45_-DMPA, a mixture of 3 g of m-PEG_45_ (1.5 mmol), 0.82 g of DMPA (2.25 mmol), and 0.02745 g of DMAP (0.22 mmol) was dissolved in 10 mL of anhydrous DCM within a 50 mL round-bottom flask. The system was sealed and purged with argon to eliminate residual oxygen. Subsequently, 0.4635 g of DCC (2.25 mmol)_,_ dissolved in 5 mL of dry DCM, was added dropwise. The reaction was conducted under constant stirring at room temperature for 24 h. The crude reaction mixture was filtered to remove dicyclohexylurea byproducts, concentrated using a rotary evaporator, and further purified by silica column chromatography using a DCM/methanol (95:5) eluent system. The purity of the final product was confirmed through thin-layer chromatography (TLC) and ^1^H-NMR spectroscopy.

**Synthesis of Diblock Copolymers PEG_x_-*b*-PVIM_y_.** All diblock copolymers were synthesized via reversible addition–fragmentation chain transfer (RAFT) polymerization. Before polymerization, vinyl imidazole (VIM) was purified using a basic alumina column to remove acidic impurities. A drop of 2,2-diphenyl-1-picrylhydrazyl (DPPH) was added to inhibit radical side reactions. A representative synthesis of PEG_45_-*b*-PVIM_100_ was carried out in a 50 mL Schlenk flask containing 0.6 g (6 mmol) of macro-PEG_45_-DMPA, 0.192 mL of VIM, 6.2 mg of AIBN (0.04 mmol), and 6.1 mL of acetic acid. The solution was degassed using three freeze-pump-thaw cycles and then heated at 70 °C for 22 h. The final product was precipitated in ether, collected, dried under vacuum, and stored.

**Conjugation of Diblock Copolymers PEG-*b*-PVIM with Chelating Agent NOTA.** A 50 mL Schlenk flask was charged with 0.2 g of PEG_45_-*b*-PVIM_100_ (2.265 × 10^−5^ mol) and an appropriate amount of maleimide-NOTA (2.265 × 10^−6^ mol, 0.1 equivalents), dissolved in dry DMF. Separately, DBU was dissolved in DMF and added to the reaction mixture. The reaction proceeded at 70 °C for 24 h. The final product was purified via recrystallization in ether, collected, dried under vacuum, and stored.

**Synthesis of ^99m^Tc Complexes**. The *fac*-[^99m^Tc(CO)_3_(H_2_O)_3_]^+^ precursor was prepared using the homemade kit, and its radiochemical purity was confirmed by reverse-phase HPLC. A solution (0.5–1.0 mL, 37–740 MBq, pH 6) of this precursor was mixed in a capped vial with 100 μg of each macro-compound [Macro-PEG_16_(^99m^Tc-1), PEG_16_-*b*-PVIM_48_(^99m^Tc-2), PEG_114_-*b*-PVIM_86_(^99m^Tc-3), PEG_114_-*b*-PVIM_48_(^99m^Tc-4), PEG_45_-*b*-PVIM_34_(^99m^Tc-5), PEG_45_-*b*-PVIM_69_(^99m^Tc-6), and Macro-PEG_45_(^99m^Tc-7)]. The mixture was incubated at 80 °C for 30 min, followed by HPLC analysis [22]. For subsequent stability and animal studies, each complex was purified by HPLC to achieve a radiochemical purity (RCP) of 99.5%. Solvents were then removed under a gentle flow of N_2_ at 40 °C, and the radiotracers were reconstituted in phosphate-buffered saline (PBS, 10 mM, pH 7.4) containing 10% ethanol.

**In Vitro Stability Studies of ^99m^Tc Complexes.** HPLC-purified samples of the ^99m^Tc complexes were kept at room temperature for up to 6 h. To assess stability, challenge experiments were conducted by adding 0.8 mL of PBS (pH 7.4) and 0.1 mL of a 0.1 M aqueous solution of either cysteine or histidine to 0.1 mL (3.7 MBq) of each ^99m^Tc complex, followed by incubation at 37 °C. Samples were collected at 1, 3, and 6 h for HPLC analysis. For serum stability tests, 0.1 mL of each tracer (3.7 MBq) was mixed with 0.4 mL of human serum and incubated at 37 °C. Non-specific serum protein binding was determined by precipitating proteins with 0.5 mL of acetonitrile, centrifuging at 5000 rpm for 5 min, and collecting the supernatant. The protein pellet was redissolved in 0.2 mL acetonitrile and centrifuged again. The combined supernatants were measured using a dose calibrator to calculate the binding percentage. Subsequently, the acetonitrile was removed by gentle heating at 40 °C under a stream of N_2_, and the residues were reconstituted in saline (with 10% ethanol) before analysis by radio-HPLC [23].

**Polymer Characterization.** Gel Permeation Chromatography (GPC). The molecular weights (MW) and molecular weight distributions (MWDs) of the synthesized copolymers were determined using GPC. A Waters 2695 system equipped with a Waters 2414 refractive index detector (Waters, Belgium) and an Ultrahydrogel linear column (10 μm, 7.8 mm × 300 mm, 1K–7M) was used. The mobile phase consisted of 0.1 M NaNO_3_ in H_2_O at a flow rate of 1 mL/min. Calibration was performed using nine narrow-distribution PEG standards (106, 390, 1010, 1490, 4070, 10,300, 19,500, 31,700, and 72,300 g/mol). The number-average molecular weight (Mn), weight-average molecular weight (Mw), and polydispersity index (Mw/Mn) were calculated.

Nuclear Magnetic Resonance Spectroscopy (NMR). ^1^H-NMR spectra of DMPA, MacroPEG-DMPA, PEG_x_-*b*-PVIM_y_ copolymers, and PEG_x_-*b*-PVIM_y_-NOTA conjugates were recorded in D_2_O, CDCl_3_, and d^6^-DMSO using Bruker Avance 300 and 500 MHz spectrometers to confirm the structure and composition.

Hydrogen ion titration. The protonation behavior of the diblock copolymers was investigated through acid–base titration. Aqueous solutions (0.01 wt%) of each copolymer were titrated with a standard 1 M NaOH solution from pH 2 to 11 and monitored using a HANNA HI991300 portable pH meter. Furthermore, the effective pK of the PVIM units was estimated as the pH at 50% ionization.

Dynamic Light Scattering (DLS). The hydrodynamic diameters of the synthesized diblock copolymers in H_2_O were measured using a Brookhaven 90Plus DLS spectrophotometer with a BI9000 correlator and a 30 mW red diode laser (673 nm) at a 90° angle. A 1 wt% polymer solution was filtered through a 0.45 μm PTFE syringe filter, allowed to settle for one hour to remove air bubbles, and analyzed using multimodal size distribution (MSD) analysis based on non-negatively constrained least squares (NNCLS).

Distribution Coefficient (D_o/w_) Determination. The lipophilicity of the ^99m^Tc complexes was determined using the shake-flask method. In brief, 10 μL (approximately 370 kBq) of the purified complex was added to a centrifuge tube containing 3 mL of a 1:1 mixture of 1-octanol and PBS (0.1 M, pH 7.4). After vortexing for 1 min at room temperature, the mixture was centrifuged at 5000 rpm for 5 min. Radioactivity from three separate 0.1 mL aliquots from both the 1-octanol and PBS layers was measured using a gamma counter. Additional partitioning of a 0.5 mL aliquot of the octanol phase was performed until consistent counts were obtained. This procedure was repeated three times, and the D_o/w_ was calculated as the ratio of counts in the octanol phase to those in the PBS phase [23].

Imaging Studies/Imaging System. Real-time dynamic imaging was performed using a specialized, mouse-sized planar scintigraphy system (γ-eye™ by BIOEMTECH, Athens, Greece). This system fuses scintigraphic data with digital photographs of the subject and employs a deep neural network to generate synthetic X-ray images for enhanced anatomical co-registration [24]. Based on position-sensitive photomultiplier tubes (PSPMTs) coupled with a CsI(Na) pixelated scintillator and a medium-energy lead collimator with parallel hexagonal holes, the detector is optimized for various SPECT isotopes. The system is well-suited for high-quality planar imaging with a field of view of 5 × 10 cm^2^ and a spatial resolution of approximately 2 mm. During imaging sessions, healthy Swiss Albino mice were maintained under isoflurane anesthesia and at a constant temperature of 37 °C. Short static scans (typically 10 min or less) were acquired at multiple time points to monitor tracer distribution over time [25]. Image post-processing and quantification were performed using the embedded visual|eyes software (BIOEMTECH, Athens, Greece).

Animal Imaging Studies. For SPECT imaging, mice received intravenous bolus injections of ^99m^Tc tracers (0.1 mL, ~10 MBq) under isoflurane anesthesia (induction at 3–5% and maintenance at 1–3%). Imaging was conducted on live animals to capture real-time biodistribution data [26]. All the biodistribution experiments were carried out in compliance with the national laws and European protocols (2010/63/EU) related to the conduct of animal experimentation. The corresponding animal experiments were approved by the Hellenic Ministry of Rural Development and Food (426573/03-04-2024).

## 3. Results and Discussion

**Figure 1:**
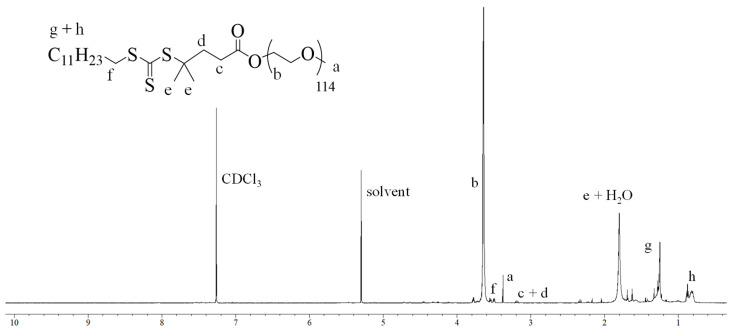
^1^H NMR spectra of MacroPEG_114_-DMPA in CDCl_3_.

**Synthesis and Characterization of Macro-PEG-DMPA.** The synthesis of the macro chain transfer agent (MacroPEG-DMPA) was carried out via Steglich esterification, a mild and efficient method that ensures high conversion rates while maintaining the integrity of sterically demanding substrates. The reaction involved the coupling of poly(ethylene glycol) methyl ether (m-PEG-OH) with 2-Dodecylthiocarbonylthio-2-methylpropanoic acid (DMPA) in the presence of dicyclohexylcarbodiimide (DCC) as the coupling agent and 4-dimethylaminopyridine (DMAP) as the catalyst. This reaction sequence resulted in the successful formation of Macro-PEG-DMPA, which was subsequently purified through multiple steps, including filtration to remove dicyclohexylurea byproducts and column chromatography using a DCM/methanol solvent system. Three different macro-CTA agents were prepared in total, MacroPEG_16_-DMPA, MacroPEG_45_-DMPA, and MacroPEG_114_-DMPA, which varied in terms of the degree of polymerization of PEG chains. The chemical structure of the obtained Macro-PEG-DMPA was confirmed via proton nuclear magnetic resonance (^1^H NMR) spectroscopy. The characteristic peaks corresponding to the ethylene oxide repeat units of PEG were observed in the region of 3.5 ppm, while the signals associated with the thiocarbonylthio moiety of DMPA were detected at approximately 1.2 and 3.3 ppm. These spectral features confirmed the successful attachment of the RAFT agent to the PEG backbone. In Figure 1, the ^1^H NMR spectrum of MacroPEG_114_-DMPA is shown.

**RAFT Polymerization and Synthesis of PEG_x_-*b*-PVIM_y_ Diblock Copolymers.** Reversible Addition–Fragmentation Chain Transfer (RAFT) polymerization was employed to synthesize amphiphilic poly(ethylene glycol)-*block*-poly(1-vinylimidazole) (PEG-*b*-PVIM) copolymers, aiming to achieve precise control over molecular weight and narrow dispersity. However, initial polymerization attempts necessitated systematic optimization of reaction parameters, including the choice of chain transfer agent (CTA), solvent, monomer concentration, and initiator-to-CTA molar ratio, to attain successful polymerization. In the initial experiment, macroPEG_16_ functionalized with 4-cyano-4-(dodecylsulfanylthiocarbonyl)sulfanyl pentanoic acid (macroPEG_16_-CDPA) served as the CTA, with DMF as the solvent. The polymerization was conducted at 70 °C, utilizing a monomer concentration of 3.0 M and an AIBN-to-CTA molar ratio of 0.625. Under these conditions, no polymerization occurred, as confirmed by ^1^H NMR analysis. This lack of polymerization aligns with previous findings indicating that DMF may not effectively stabilize propagating radicals during RAFT polymerization of VIM [12].

Subsequently, glacial acetic acid was selected as an alternative solvent due to its dual role in stabilizing propagating radicals and preventing undesired side reactions of the imidazole moieties [17]. In the second experiment, macroPEG_16_-CDPA was again employed as the CTA, with acetic acid as the solvent. The polymerization conditions included a monomer concentration of 2.0 M and an AIBN-to-CTA molar ratio of 1.0. Despite these adjustments, no polymerization was observed, suggesting that the selected CTA and initiator ratio were still unsuitable for effective polymerization. To investigate the effect of the CTA structure, the third experiment utilized CDPA without the macroPEG chain, while retaining acetic acid as the solvent. A monomer concentration of 2.5 M and an AIBN-to-CTA molar ratio of 0.5 were tested. Under these conditions, a monomer conversion of 61% was achieved, confirming that polymerization could proceed in the absence of the macroPEG segment. In the fourth experiment, the same conditions as the third were applied, except that macroPEG_16_-CDPA was reintroduced as the CTA. Despite maintaining a monomer concentration of 2.5 M and an AIBN-to-CTA molar ratio of 0.5, a significant decrease in monomer conversion (15%) was observed. The lower monomer conversion in the presence of macroPEG_16_-CDPA is likely due to steric hindrance and reduced reactivity caused by the bulky PEG_16_ segment, which may limit the efficient transfer of the growing polymer chain between propagating radicals and the CTA.

In the fifth experiment, polymerization was performed using macroPEG_16_-CDPA in acetic acid with an increased monomer concentration of 4.0 M and an AIBN-to-CTA molar ratio of 0.625. Once again, no polymerization was detected, reinforcing the hypothesis that the selected CTA and reaction conditions were not optimal for efficient polymerization.

Given the limited success with the aforementioned CTAs, a new CTA was introduced in the subsequent phase of optimization. In the final experiment, PEG_16_ functionalized with 2-dodecylthiocarbonylthio-2-methylpropanoic acid (macroPEG_16_-DMPA) was employed as the CTA, maintaining a monomer concentration of 2.5 M and an AIBN-to-CTA molar ratio of 0.625. Under these conditions, a monomer conversion of 95% was achieved, indicating that macroPEG_16_-DMPA is a more effective CTA for the RAFT polymerization of VIM. Table 1 summarizes the experimental conditions and outcomes of the polymerization attempts.

**Figure 2:**
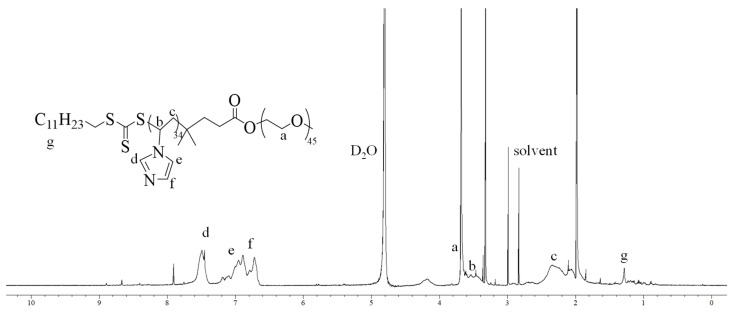
^1^H NMR spectrum of PEG_45_-*b*-PVIM_34_ in D_2_O.

After identifying the optimal conditions for VIM polymerization, the synthesis of five diblock copolymers with varying PEG-to-VIM molar ratios and different polymer chain lengths was carried out. The copolymers PEG_16_-*b*-PVIM_48_, PEG_45_-*b*-PVIM_69_, PEG_114_-*b*-VIM_86_, PEG_45_-*b*-PVIM_34_, and PEG_114_-*b*-VIM_48_ were successfully synthesized under these optimized conditions. The successful preparation of these copolymers was confirmed via ^1^H NMR spectroscopy. Figure 2 presents the ^1^H NMR spectrum of PEG_45_-*b*-PVIM_34_ in D_2_O. As observed in the spectrum, the characteristic aromatic peaks of the PVIM block appear in the region of 6.5–7.5 ppm, confirming the presence of the imidazole moieties within the polymer backbone. This further validates the successful polymerization and formation of the diblock copolymers.

**(Co)polymer Molecular Weights and Compositions.** In this study, five PEG-*b*-PVIM diblock copolymers with varying chain lengths and compositions were synthesized under optimized RAFT polymerization conditions. The monomer conversion rates, determined via ^1^H NMR spectroscopy, and the molecular weights of the resulting copolymers are summarized in Table 2.

As shown in Table 2, monomer conversion rates ranged from 69.4% to 95.7%, with higher degrees of polymerization correlating with increased steric hindrance, which may have impeded monomer incorporation. Notably, the experimental M_n_ values obtained from GPC were consistently lower than the theoretical M_n_ values calculated based on monomer conversion determined by ^1^H NMR. This difference can be attributed to interactions between the copolymers and the GPC column, as well as potential micelle formation during analysis. Such interactions may lead to delayed elution, polymer aggregation, or altered hydrodynamic volume, ultimately resulting in underestimated molecular weights. Similar findings have been reported in the literature, where polymer–column interactions and self-assembly behaviors have caused GPC to underestimate molecular weights [27,28,29].

The polydispersity indices (*Ð*) of the diblock copolymers, as determined by GPC, were all ≤1.3, indicating a narrow molecular weight distribution and a well-controlled polymerization process. These results suggest that factors such as polymer solubility, column interactions, and potential micellization may influence molecular weight determination by GPC.

Table 2 also presents the PVIM content of the diblock copolymers as calculated from ^1^H NMR spectroscopy. The experimental values closely matched the theoretical values, confirming that the polymerization of VIM was well-controlled. However, in all cases, the theoretical values calculated from initial monomer loading were higher than the experimental values, likely due to termination reactions occurring during polymerization.

Despite these variations, the successful synthesis of PEG_x_-*b*-PVIM_y_ diblock copolymers with different PEG-to-PVIM ratios was confirmed via ^1^H NMR spectroscopy, further demonstrating the controlled nature of the polymerization process.

**Effective pK Values.** The effective pK values of the imidazole units in the synthesized diblock copolymers were determined using potentiometric titration. These values were calculated by analyzing the titration curves, where two asymptotic lines were constructed, and a perpendicular line was drawn to identify the equivalence point. The pH corresponding to half the NaOH volume (V) added at the equivalence point was recorded as the pK value.

As shown in Table 3, the imidazole units in all copolymers exhibited pK values of approximately 4.2, irrespective of polymer composition. These values were lower than the reported pK of the free VIM monomer (6.07) [30], which is consistent with the behavior of weak polyelectrolytes. This shift can be attributed to electrostatic repulsions between ionized imidazole groups along the polymer backbone, which hinder further ionization and result in a lower pK compared to the monomeric form.

Despite the deprotonation of the imidazole groups at physiological pH, the resulting copolymers remained water-soluble, as confirmed by acid–base titration.

**Solution Micellization.** To evaluate the self-assembly behavior of PEG_x_-*b*-PVIM_y_ diblock copolymers in aqueous solution, dynamic light scattering (DLS) measurements were performed to determine the hydrodynamic diameters (D_h_) and polydispersity index (PDI) of the resulting aggregates. The thus-determined hydrodynamic diameters are listed in Table 4. The same table shows the upper limit of the size of the micelles of the diblock copolymer calculated for fully stretched chains in spherical micelles, calculated by multiplying the total DP of the linear copolymers times 0.252 nm, the contribution of one monomer repeating unit [31], then multiplying by two to convert the maximum micelle radius to the maximum diameter.

The effective hydrodynamic diameters were found to be 428.2 nm for PEG_114_-*b*-VIM_86_, 222 nm for PEG_114_-*b*-PVIM_48_, 243.2 nm for PEG_45_-*b*-PVIM_69_, and 370.4 nm for PEG_45_-*b*-PVIM_34_. The monomodal size distributions and PDI values below 0.2, and the higher values of experimental hydrodynamic diameters than the upper limit of the size of the micelles, suggest that relatively uniform aggregates were formed in all cases.

The formation of large aggregates instead of small micelles is consistent with previous studies on PEG-based block copolymers [32]. Similar systems, such as PEG-*b*-polyvinylpyrrolidone (PEG-*b*-PVP) [33], have been reported to self-assemble into nanoparticles or large aggregates in water, depending on the block ratio and polymer concentration. In the case of PEG_x_-*b*-VIM_y_, the relatively high D_h_ values observed in our study suggest that these copolymers form aggregates through hydrophobic interactions between VIM blocks, which are not fully shielded by the hydrophilic PEG chains.

The monomodal size distributions observed in all samples indicate that the aggregates formed are relatively uniform, which differs from the behavior of some PEGylated diblock copolymers that exhibit bimodal or trimodal distributions depending on concentration and solvent conditions [33]. This suggests that PEG_x_-*b*-PVIM_y_ copolymers may have a well-defined self-assembly pathway in aqueous media, where a single population of aggregates dominates under the studied conditions.

Overall, these findings indicate that PEG-*b*-VIM copolymers self-assemble into large aggregates in water rather than well-defined micelles, with the aggregate size influenced by the PEG-to-VIM ratio.

**Conjugation of Diblock Copolymers PEG-*b*-PVIM with Chelating Agent NOTA.** Following the successful synthesis of PEG_x_-*b*-PVIM_y_ diblock copolymers, their conjugation with the maleimide-functionalized NOTA chelating agent was performed to facilitate radiolabeling for potential in vivo biodistribution studies. The conjugation reaction was carried out through a two-step aminolysis-click reaction mechanism. Initially, aminolysis of the trithiocarbonate end-group was performed using DBU, which acted as a nucleophile, attacking the electrophilic carbon of the trithiocarbonate moiety, leading to the formation of a free thiol group at the end of the polymer chain. This newly formed thiol then underwent a Michael addition (“click” reaction) with the electron-deficient maleimide functionality of NOTA, enabling efficient and selective bioconjugation under mild conditions. To ensure the removal of unreacted reagents and reaction byproducts, the conjugated PEG_16_-*b*-VIM_48_-NOTA copolymers were purified via precipitation in cold diethyl ether. The purified product was then collected by filtration, dried under vacuum, and stored for further characterization.

**Figure 3:**
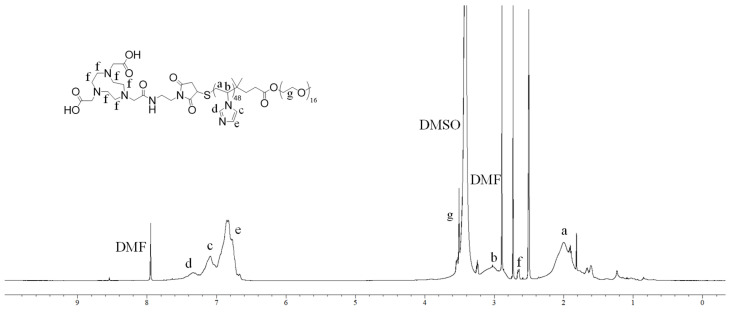
^1^H NMR spectrum of PEG_16_-*b*-PVIM_48_ in DMSO-d^6^.

The successful conjugation of NOTA to PEG_16_-*b*-PVIM_48_ was confirmed via ^1^H NMR spectroscopy, as shown in Figure 3. The spectrum exhibited a distinct peak at 3.5 ppm, corresponding to the ethylene oxide protons of the PEG segment, while the aromatic protons of the imidazole moiety appeared around 7.0 ppm. Additionally, the aliphatic protons of NOTA were detected at approximately 2.8 ppm, providing clear evidence of successful modification.

This post-polymerization functionalization enhances the biomedical applicability of the synthesized diblock copolymers, enabling their use in radiolabeling and real-time tracking of nanocarriers in biological systems.

**Radiolabeling.** Radiolabeling was achieved using the ^99m^Tc(CO)₃ tricarbonyl core. The precursor, *fac*-[^99m^Tc(CO)_3_(H_2_O)_3_]^+^, was synthesized in-house by direct addition of ^99m^TcO_4_^−^ to a sealed vial containing CO gas and NaBH_4_ as a reducing agent, followed by heating at 95 °C for 30 min. The reaction mixture was then adjusted to pH 7. Quality control by HPLC confirmed the efficient formation of the precursor, with a radiochemical purity exceeding 97%.

The macrocyclic compounds were radiolabeled with *fac*-[^99m^Tc(CO)_3_(H_2_O)_3_]^+^ at low ligand concentrations, under heating at 80 °C for 30 min. HPLC analysis of the reaction mixtures revealed single peaks corresponding to each complex (^99m^Tc-1 to ^99m^Tc-7), eluting at 8–10 min, clearly separated from the precursor (3.5 min) and free pertechnetate (2.8 min). For clarity, the following labels are used to identify the radiolabeled compounds: Macro-PEG_16_ (^99m^Tc-1), PEG_16_-*b*-PVIM_48_ (^99m^Tc-2), PEG_114_-*b*-PVIM_86_ (^99m^Tc-3), PEG_114_-*b*-PVIM_48_ (^99m^Tc-4), PEG_45_-*b*-PVIM_34_ (^99m^Tc-5), PEG_45_-*b*-PVIM_69_ (^99m^Tc-6), and Macro-PEG_45_ (^99m^Tc-7).

HPLC purification was performed to remove excess ligands and ensure high radiochemical purity before further in vitro and in vivo evaluation. Stability studies demonstrated that the radiotracers remained intact in their formulation for up to 6 h. Competitive incubation with histidine and cysteine—amino acids known to strongly coordinate the *fac*-[^99m^Tc(CO)_3_]^+^ core—confirmed the high stability of the complexes, with >95% of the radiotracers remaining intact (Table 5). In addition, incubation in human serum showed excellent stability over time. LogD_7.4_ values indicated moderate lipophilicity (1.1–1.8).

## 4. Preliminary Imaging and Biodistribution

**Figure 4:**
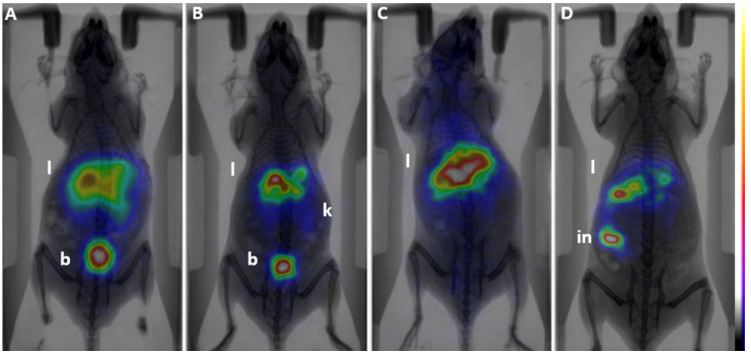
Two-dimensional imaging after intravenous injection of the ^99m^Tc-1 (**A**,**B**) and ^99m^Tc-2 (**C**,**D**) in healthy Swiss Albino mouse. (**A**,**C**): Static γ-images (5 min duration) at 5 min p.i. (**B**,**D**): Static γ-images (5 min duration) at 1 h p.i. The color bar indicates the accumulation level (i.e., white being the highest and purple the lowest). Liver (l); Kidneys (k); Intestines (in); Bladder (b).

Preliminary imaging studies have revealed distinct excretion patterns between the two radiotracers under investigation (Figure 4). ^99m^Tc-1 demonstrated rapid urinary clearance, with progressive accumulation in the bladder over time, and moderate hepatic retention, suggesting primary elimination via glomerular filtration. This observation aligns with established knowledge that small polyethylene glycol (PEG)-based molecules are predominantly excreted through the renal pathway due to their size and hydrophilicity.

In contrast, ^99m^Tc-2 exhibited significant initial liver uptake, followed by increasing intestinal accumulation, indicative of hepatobiliary excretion. Compared to ^99m^Tc-1, this tracer showed enhanced retention in the liver and spleen, which may reflect increased uptake by the reticuloendothelial system (RES). The larger hydrodynamic diameter of ^99m^Tc-2 likely contributes to its slower clearance and preferential accumulation in macrophage-rich tissues. Notably, renal excretion was minimal for ^99m^Tc-2, as evidenced by low bladder signal intensity. These findings are consistent with studies indicating that larger PEGylated compounds and nanoparticles tend to evade renal filtration, leading to prolonged circulation times and increased hepatic and splenic uptake [34]. In both tracers, brain penetration was minimal (~2%, ID), indicating limited permeability across the blood–brain barrier. Additionally, background activity in muscle and other soft tissues was low, suggesting favorable biodistribution profiles for imaging applications.

These preliminary results suggest that the incorporation of the vinylimidazole polymer in ^99m^Tc-2 significantly alters its pharmacokinetics, shifting the primary excretion pathway from renal (as observed with ^99m^Tc-1) to hepatobiliary routes. This shift may be attributed to the increased molecular size and altered physicochemical properties imparted by the polymer. Similar observations have been reported in the literature, where modifications leading to increased molecular weight and changes in hydrophilicity result in altered biodistribution and excretion pathways [35].

## 5. Conclusions

In this work, a series of PEG_x_-*b*-PVIM_y_ diblock copolymers were successfully synthesized via RAFT polymerization and systematically characterized. The polymerization conditions were optimized to achieve high monomer conversion and controlled molecular weight distributions. The copolymers exhibited pH-dependent ionization behavior, with effective pK values lower than that of the free monomer, indicative of weak polyelectrolyte characteristics. Additionally, post-polymerization modification through aminolysis enabled the conjugation of a NOTA chelator, facilitating radiolabeling for potential in vivo tracking. The successful functionalization was confirmed by ^1^H NMR spectroscopy. While drug loading was beyond the scope of this study, future research may investigate the broader utility of these copolymers in biomedical applications, including as carriers for diagnostic agents or targeted delivery systems.

## Figures and Tables

**Table 1:** Results of polymerizations performed for the optimization of the synthesis of the PEG_x_-*b*-VIM_y_ diblock copolymers. The temperature in all polymerizations was kept constant at 70 °C.

Polymer	VIM Conv. (%) ^a^	Polymerization Time (h)	Solvent	$\frac{\left[I\right]}{\left[CTA\right]}$	CTA	Monomer Concentration (M)
PVI_75_	0	24	DMF	0.625	MacroPEG_16_-CDPA	3
PVI_75_	0	24	AcOH	1	MacroPEG_16_-CDPA	2
PVI_100_	61	24	AcOH	0.5	CDPA	2.5
PVI_100_	15	24	AcOH	0.5	MacroPEG_16_-CDPA	2.5
PVI_100_	0	24	AcOH	0.5	MacroPEG_16_-CDPA	2.5
PVI_100_	0	24	AcOH	0.625	MacroPEG_16_-CDPA	4
PVI_100_	95	24	AcOH	0.122	MacroPEG_16_-DMPA	4.5

The initiator used for all the polymerization was AIBN; ^a^ monomer conversion was calculated via ^1^H-NMR.

**Table 2:** Molecular weight, composition, and monomer conversion characteristics of the polymers synthesized in this study.

No.	Polymer Structure ^a^	MW Theory ^b^	GPC			Mon. Conv. ^c^	Theoretical ^d^ VIM (mol%)	^1^H NMR ^e^
			*M*_p_	*M*_n_	*Ð*			VIM (mol%)
1	PEG_16_-*b*-PVIM_48_	5647	-	-	-	95	86	75
2	PEG_45_-*b*-PVIM_69_	8763	2079	2315	1.11	69	82	71
3	PEG_45_-*b*-PVIM_34_	5947	1887	2159	1.14	78	70	71
4	PEG_114_-*b*-VIM_86_	13,361	4610	6059	1.31	86	65	50
5	PEG_114_-*b*-VIM_48_	9789	4829	6371	1.31	96	48	46

^a^ The DP of VIM was calculated based on monomer conversion determined by ^1^H NMR. ^b^ Theoretical molecular weights were calculated from the DPs given in the polymer structure column. ^c^ Conversion of monomer polymerized determined by ^1^H NMR. ^d^ The theoretical VIM content was calculated based on the initial monomer loading during polymerization. ^e^ Experimental values calculated from the ^1^H NMR spectrum of the dried polymer.

**Table 3:** pK values of PEG_x_-*b*-PVIM_y_ copolymers.

No.	Polymer Structure	pK	^1^H NMR
			VIM (mol%)
1	PEG_45_-*b*-PVIM_69_	4.1	71
2	PEG_45_-*b*-PVIM_34_	4	71
3	PEG_114_-*b*-VIM_86_	4.05	50
4	PEG_114_-*b*-VIM_48_	4.20	46

**Table 4:** Hydrodynamic diameters of the PEG_x_-*b*-PVIM_y_ diblock copolymers in H_2_O.

Polymer Structure	Theoretically Maximum D_h_ (nm)	H_2_O	
		Experimental D_h_ (nm)	PDI
PEG_45_-*b*-PVIM_69_	57	243.2	0.166
PEG_45_-*b*-PVIM_34_	39	370.4	0.041
PEG_114_-*b*-VIM_86_	101	428.2	0.141
PEG_114_-*b*-VIM_48_	82	222.0	0.169

**Table 5:** HPLC retention times (t_R_) of ^nat^Re-complexes co-injected with ^99m^Tc tracers, along with radiochemical yield (RCY), log D_7.4_ values, stability in L-cysteine (6 h), L-histidine (6 h), human serum (6 h), and non-specific human serum protein binding (6 h).

	^99m^Tc-1	^99m^Tc-2	^99m^Tc-3	^99m^Tc-4	^99m^Tc-5	^99m^Tc-6	^99m^Tc-7
Tracer t_R_ (min)	8.0	8.5	8.3	9.2	9.7	9.0	8.2
RCY (%)	72 ± 10	78 ± 5	75 ± 15	82 ± 10	88 ± 15	67 ± 5	75 ± 10
Log D_7.4_	1.1 ± 0.2	1.8 ± 0.2	1.3 ± 0.1	1.5 ± 0.1	1.5 ± 0.2	1.2 ± 0.1	1.2 ± 0.3
Cys (%) ^a^	>97	>97	>97	>97	>97	>97	>97
Hist (%) ^a^	>97	>97	>97	>97	>97	>97	>97
Serum (%) ^a^	80 ± 2	84 ± 5	82 ± 4	80 ± 7	84 ± 5	88 ± 4	82 ± 2
Protein Binding (%) ^a^	10 ± 3	7 ± 3	8 ± 2	6 ± 3	4 ± 2	5 ± 1	5 ± 2

All values are the mean ± SD of three independent determinations. ^a^ % Radioconjugate intact at 6 h.

## Data Availability

The original contributions presented in this study are included in the article. Further inquiries can be directed to the corresponding author.
